# Fast Identification Method for Screening Bacteria from Faecal Samples Using Oxford Nanopore Technologies MinION Sequencing

**DOI:** 10.1007/s00284-023-03201-7

**Published:** 2023-02-09

**Authors:** Ana Sofia G. Borges, Meghna Basu, Erik Brinks, Corinna Bang, Gyu-Sung Cho, John F. Baines, Andre Franke, Charles M. A. P. Franz

**Affiliations:** 1 Department of Microbiology and Biotechnology, Max Rubner-Institut, Federal Research Institute for Nutrition and Food, Hermann-Weigmann-Straße 1, 24103 Kiel, Germany; 2 Section of Evolutionary Medicine, Institute for Experimental Medicine, Christian-Albrechts-University Kiel, UKSH, Campus Kiel, Michaelisstraße 5, 24105 Kiel, Germany; 3 Max Planck Institute for Evolutionary Biology, August-Thienemann-Straße 2, 24306 Plön, Germany; 4 Institute of Clinical Molecular Biology, Christian Albrechts University of Kiel, Rosalind-Franklin-Straße 12, 24105 Kiel, Germany

## Abstract

Most bacterial identification methods require extensive culturing, strain purification and DNA extraction protocols. This leads to additional expenses and time lags when isolating specific bacteria from complex microbiological ecosystems. This study aimed to develop a fast and robust method for identification of lactobacilli, bifidobacteria and *Bacteroides* in human faecal samples. Bacteria from faecal samples were cultured anaerobically on selective media. Sonication-based DNA extraction was performed, followed by almost complete 16S rRNA gene polymerase chain reaction amplification and MinION sequencing with the Flongle adapter. Sequence analysis was performed using NanoCLUST, while RStudio was used for graphics. For 110 of the 125 colonies investigated, 100% of reads were attributed to a single species, while the remaining 15 colonies consisted of mixtures of up to three different species. The proposed bacterial identification method is advantageous for isolating particular bacteria for which there are no exclusively selective media, as it avoids lengthy colony purification and DNA purification methods, and yields a quick colony identification with high accuracy. Therefore, this method can be used for directly screening for pure cultures of target microorganisms and is suitable for the identification of bacteria in culturomics studies.

**Supplementary Information:**

The online version contains supplementary material available at 10.1007/s00284-023-03201-7.

## Introduction

Non-selective microbiological growth media can support the growth of a wide variety of microorganisms by supplying a rich nutrient source. On the other hand, selective media often include growth inhibiting substances that select for the types of bacteria that can grow in the presence of these and inhibit or eliminate the growth of unwanted microbiota [1]. The use of selective media is often necessary for targeted isolation of specific bacteria that occur in complex microbiological ecosystems such as food, plants, soil, medical samples and the gastrointestinal tract. The drawback of selective media is often the lack of exclusive selectivity, meaning that for a group of microorganisms, the isolates obtained from the media are not always those targeted by the selective conditions.

For example, the selective medium de Man, Rogosa and Sharpe (MRS) [2] is widely used for the isolation of lactic acid bacteria (LAB), but other Gram-positive, acid-tolerant bacteria such as bifidobacteria and certain *Bacillus* spp. can grow on this medium, as can some yeasts and moulds [3]. Furthermore, many *Bifidobacterium* media available rely on the MRS agar base with various additives (e.g. cysteine hydrochloride, mupirocin, lithium chloride or raffinose) that make it selective for these bacteria. Lithium propionate MRS agar (LP-MRS) was previously shown to support the growth of bifidobacteria, while LAB such as *Lactobacillus delbrueckii* and *Streptococccus thermophilus* were inhibited [4]. Another example of selective media is the *Bacteroides*-specific *Bacteroides fragilis* bile-esculin (BBE) agar for the isolation of *Bacteroides* spp., which contains the selective agents gentamicin (which inhibits facultative anaerobes) and bile (which inhibits most Gram-positive bacteria and anaerobic microorganisms other than the *Bacteroides fragilis* group). However, some *Enterococcus, Klebsiella* and *Fusobacterium* strains may grow on BBE agar, though they may be distinguished by different colony characteristics [5].

To obtain pure cultures for storage in culture collections, characterization and comparisons of strains, there is still a need for purification of isolates which is generally done by repeated rounds of isolating colonies, growing these in liquid media and streaking these out for purity onto agar media. This is both time consuming and costly in terms of media and equipment. The isolation from agar media then usually requires further identification of the strains either by 16S rRNA gene sequencing, or other suitable methods such as matrix-assisted laser desorption ionization time-of-flight (MALDI-TOF) mass spectrometry (MS) strain typing [6].

In recent years, considerable attention has been given to culturomics, which is based on a better understanding of the bacterial environments and adaptation of this knowledge to optimize growth media by, e.g. adding new elements into the culture media, isolating microcolonies or increasing the number of culture conditions [1]. It has been particularly useful for the study of the gut microbiota together with metagenomic studies, which have unravelled a large variety of gut-associated microorganisms that had previously not been described, due to their failure to grow on laboratory media [6]. Such culturomics studies generally rely on MALDI-TOF or 16S rRNA gene polymerase chain reaction (PCR) to identify bacteria [6, 7].

This study aimed to develop a method which would allow for the fast isolation and identification of bacterial colonies obtained from human faecal samples. This method relied on the use of selective media to isolate LAB, bifidobacteria and *Bacteroides* spp. present in faecal samples. Colonies on plates were picked, grown in broth media and directly identified by using Oxford Nanopore Technologies (ONT) sequencing and bioinformatic analyses, which also allowed an insight into the purity of the isolates. Depending on the application (e.g. culturomics, genomics), this method can be advantageous in terms of time and costs when compared to alternatives.

## Materials and Methods

### Isolation of Bacteria from Human Faeces

Healthy blood donors from Northern Germany were recruited by mail and asked to provide stool samples in diverse buffer solutions. Transport of samples was done by mail within 24 h after sample collection. Immediately after their arrival at the Microbiome laboratory of the Institute of Clinical Molecular Biology, the stool samples were aliquoted and stored at − 80 °C until further processing. The study was conducted in accordance with the Declaration of Helsinki, and ethical approval was granted by the ethics committee at Kiel University (AZ A103/14, D590/22). Six randomly chosen faecal samples from this study collective were transferred to an A45 anaerobic workstation (Don Whitley Scientific, Meintrup, Herzlake, Germany) with an atmosphere consisting of 10% hydrogen, 10% carbon dioxide and 80% nitrogen at 37 °C. Inside the anaerobic chamber, the faecal samples were thawed, and 100 µl of faeces were added to 900 µl of quarter-strength Ringers solution (QSRS) (Merck, Darmstadt, Germany) and diluted in a ten-fold dilution series in QSRS. 100 µl aliquots of these dilutions were plated out onto MRS agar (Merck), LP-MRS agar (MRS containing 3 g/L sodium propionate and 2 g/l lithium chloride) [4] and BBE agar [5] to isolate LAB, bifidobacteria and *Bacteroides* spp., respectively. All dilutions were plated out by spread plating. The QSRS and all culture media plates were previously incubated for 24 h in the anaerobic chamber to allow to equilibrate to anaerobic conditions. Plates were incubated in the anaerobic chamber at 37 °C for 48 h, after which colonies from plates of the highest dilutions that showed well-separated single colonies were randomly picked for isolating bacteria. Ten colonies were picked per medium per donor. Single colonies were transferred to Hungate tubes containing 10 ml of anaerobic broth media of the same type as the plate from which the colonies were picked for lactobacilli and *Bifidobacteria,* or in supplemented Brain Heart Infusion Supplemented (BHIS) medium for *Bacteroides* [8]. The broth media used for inoculation were previously prepared anaerobically by flushing with N_2_ gas at 0.5 bar in the Hungate tubes. After autoclaving, the Hungate tubes with broth media were incubated for 24 h in the anaerobic chamber to equilibrate to anaerobic conditions. Single colonies were incubated anaerobically for 2–5 days at 37 °C depending on the turbidity of the medium, so that only well-grown cultures were used for DNA isolation.

### DNA Isolation and 16S rRNA Gene PCR Amplification

A 10 µl aliquot of each culture was removed after incubation in the anaerobic chamber and added to 990 µl of sterile bi-distilled water in Eppendorf cups. The bacterial cultures were placed in an Emmi-H40 ultrasonicator (EMAG AG, Mörfelden-Walldorf, Germany) filled with water and ice, and sonicated at a half-wave operating frequency of approx. 40 kHz (total power 440 W, ultrasonic power max. 240 W) for 2.5 min, after which samples were put on ice for 1 min [9]. This procedure was repeated six times. Ice was regularly added to the ultrasonicator as it melted during the sonication process. After the last sonication cycle, the sample was kept on ice for 5 min to allow the debris to settle, and 10 µl of supernatant was then collected to serve as DNA template for 16S rRNA gene amplification by PCR. The PCR reaction was performed in a 50 µl volume and consisted of 10 µl of template DNA, 2.5 µl of each forward and reverse primers (final concentration 0.5 µM), 6 µl of dNTPs (final concentrations 600 µM each), 10 µl of 5 × *Taq* polymerase buffer and 1 unit of Q5 high-fidelity DNA polymerase (New England Biolabs, Massachusetts, United States). The primers used were specific for amplification of the almost complete 16S rRNA gene and included the modified forward 27F (5′-AGR GTT TGA TCM TGG CTC AG-3′) and the 1492R (5′-TAC CTT GTT ACG ACT T-3′) primers [10]. A set of barcoded 16S rRNA gene primers were used to directly apply the PCR products for ONT sequencing. Therefore, barcodes and spacer sequences were added to the 27F and 1492R primers as described elsewhere [11].

DNA was amplified by PCR (initial denaturation, 94 °C for 3 min; 32 cycles of 94 °C for 30 s, 56 °C for 30 s and 72 °C for 1 min 10 s, followed by a final extension at 72 °C for 5 min). 5 µl of each PCR product was subjected to electrophoresis on a 1.5% TAE [Tris–acetate- ethylenediaminetetraacetic acid (EDTA)] gel in 1 × TAE buffer for 1 h at 100 V and bands were checked for products of the expected size (ca. 1500 bp) under UV light using the GelRed Nucleic Acid Stain (Merck). The PCR products were purified using the Mag-Bind® TotalPure NGS Kit (Omega) with a ratio of 1:1 (v/v). Purified PCR products were then quantified using the Qubit™ dsDNA BR Assay Kit (Invitrogen™) and a Tecan Spark multimode microplate reader (Tecan) followed by equimolar pooling of the samples.

### ONT MinION 16S rRNA Gene Sequencing and Data Analysis

200 ng of the equimolar pool was used for sequencing on an ONT MinION Flongle (ONT, United Kingdom) following the “Genomic DNA by Ligation (SQK-LSK109)” protocol for Flongle flow cells (Version: GDE_9063_v109_revAI_14Aug2019). These flow cells allow for the sequencing of up to 96 samples per run. The final library was quantified using the Qubit™ dsDNA HS Assay Kit (Invitrogen™) and a Qubit 3.0 Fluorometer (Invitrogen™). A final library concentration of 20 fmol was loaded on the Flongle flow cell and sequenced for 24 h.

Once the raw data files were obtained, basecalling was done using the GPU Guppy Basecalling Software (version 3.4.4) with an nvidia RTX 2080. Q-score was assessed using pycoQC (v2.5.2) and was between 8.57 and 10.49. The resulting fastq files were demultiplexed using Porechop (v0.2.4) with customized barcodes [11]. The demultiplexed files can be found in the BioProject database with the accession number PRJNA914123. Q-score was checked using fastqc (v0.11.9) and Geneious Prime (2023.0.1) and values varied between Q13.7 and Q19. The demultiplexed fastq files were further analysed using NanoCLUST (v1.0dev) with standard clustering step parameters [12], and species identification of resulting consensus sequences was verified with EZTaxon [13]. RStudio (R 4.2.0) and the ggplot2 package were used to graphically represent the resulting taxonomic classification [14, 15].

### Illumina Whole Genome Sequencing (WGS) and Identity Confirmation

In order to check the species identification obtained with our 16S rRNA gene-based method at the whole genome level, we randomly selected four isolates (10670603MRS5, 10685605MRS4, 10693850MRS2, and 10693850MRS7) and sequenced their genomic DNA using Illumina short-read sequencing. Isolates 10670603MRS5 and 10693850MRS7 were sequenced using the Illumina TruSeq Nano DNA LT library Preparation Kit (Illumina, Munich, Germany), while isolates 10685605MRS4 and 10693850MRS2 were sequenced using the Illumina DNA Prep Tagmentation Kit (Illumina) according to the manufacturer’s protocol. All isolates were sequenced on an Illumina MiSeq sequencer using the MiSeq Reagent Nano Kit v2 (Illumina) (500-cycles) for paired end sequencing with 2 × 251 cycles according to the manufacturer’s instructions, but on separate runs for the different protocols.

The raw WGS data were trimmed using the Trimmomatic pipeline (v. 0.32; parameters: Phred = 33, sliding window = 4:15, leading = 3 and minlen = 45) [16] and de novo assembly was performed using the SPAdes pipeline (v. 3.10.0; parameters: –pe, kmer: 21, 33, 55, 77; –careful) [17]. After de novo assembly, contig sequences that were shorter than 500 bp or contaminated with spiked PhiX sequence were removed using the BBDuk pipeline [18]. The average nucleotide identity (ANI) and the digital DNA-DNA hybridization (dDDH) of WGS data were performed using the OrthoANI pipeline (v. 1.2) with USEARCH tool as default parameters [19] and the Genome-to Genome Distance Calculator using formula 2, respectively [20, 21].

## Results and Discussion

**Fig. 1:**
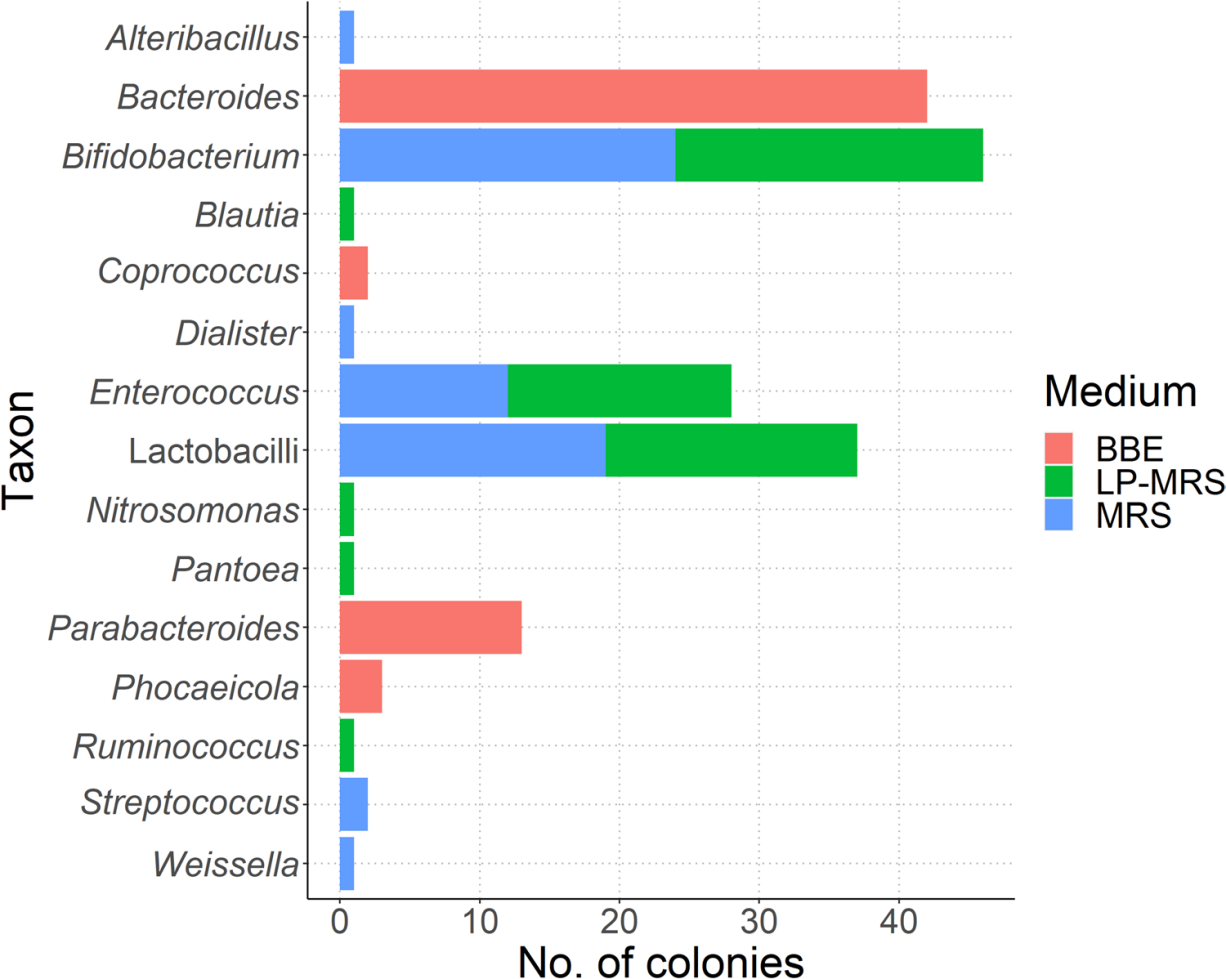
rRNA gene-based identification of 180 colonies from faecal samples according to culture media. In case of impure colonies, only the taxon with the highest number of sequence reads was included. Red colour corresponds to colonies cultured in BBE medium, green for colonies cultured in LP-MRS and blue for colonies cultured in MRS (Color figure online)

The results of the sequencing of 180 isolates (60 for each agar medium) are shown in Fig. 1, according to the agar medium the colonies were isolated from. Of the 60 colonies isolated from MRS agar medium, the majority could be identified as *Bifidobacterium* spp. (*n* = 24), lactobacilli (different genera based on the reclassification of the genus *Lactobacillus* [22]) (*n* = 19) and *Enterococcus* spp. (*n* = 12), while only few were identified as *Streptococcus* (*n* = 2)*, Weissella* (*n* = 1)*, Alteribacillus* (*n* = 1) and *Dialister* (*n* = 1). Regarding the LP-MRS agar used to isolate bifidobacteria, the majority of the colonies could be identified as *Bifidobacterium* (*n* = 22), lactobacilli (*n* = 18) and *Enterococcus* (*n* = 16), while one of each could be identified as *Blautia, Nitrosomonas, Pantoea* and *Ruminococcus.* In case of the BBE agar for isolation of *Bacteroides* spp., the bacteria were identified as *Bacteroides* (*n* = 42), *Parabacteroides* (*n* = 13), *Phocaeicola* (*n* = 3) and *Coprococcus* (*n* = 2) (Fig. 1).

**Fig. 2:**
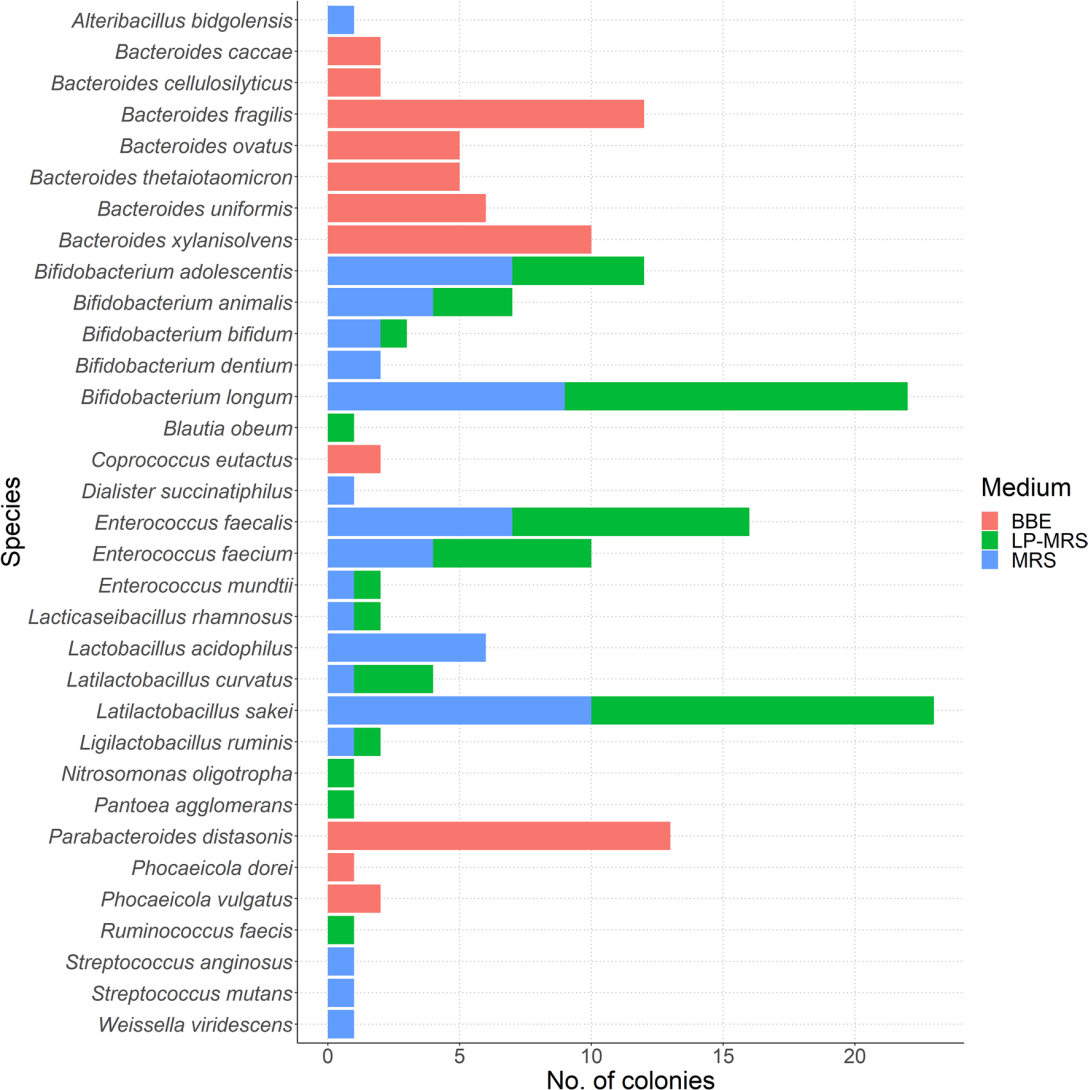
Species-level 16S rRNA gene-based identification of 180 colonies from faecal samples according to culture media. In case of impure colonies, only the species with the highest number of sequence reads was included. Red colour corresponds to colonies cultured in BBE medium, green to colonies cultured in LP-MRS and blue to colonies cultured in MRS (Color figure online)

For analysis of the predominant species, in the case of impure colonies in which more than one genus/species occurred, only the species with the highest number of sequence reads were included. The predominant *Bacteroides* (*Ba.*) spp. isolated from BBE agar were *Ba. fragilis* and *Ba. xylanisolvens*, while *Ba*. *uniformis*, *Ba*. *ovatus* and *Ba. thetaiotamicron* were also isolated frequently. Only a few isolates of *Ba. caccae* and *Ba. cellulosilyticus* could be obtained (Fig. 2). Among the lactobacilli (*L.*), the genera *Lactobacillus, Lacticaseibacillus, Latilactobacillus* and *Ligilactobacillus* were identified. Interestingly, *L. acidophilus* could only be isolated from MRS medium (Fig. 2), while the other lactobacilli species could be isolated from both MRS and LP-MRS. *L. sakei* was the most frequently isolated, while *L. rhamnosus, L. acidophilus, L. curvatus* and *L. ruminis* could be isolated less frequently. The *Enterococcus* (*E.*) spp. isolated from both LP-MRS and MRS included mainly *E. faecalis* and *E. faecium*, while bacteria from two colonies were identified as *E. mundtii*. Bifidobacteria could be isolated from both MRS and LP-MRS in most cases, except for *Bifidobacterium* (*Bf.*) *dentium*, for which only few isolates were obtained. The most frequently isolated bifidobacteria were *Bf. longum*, *Bf. adolescentis* and *Bf. animalis,* while *Bf. bifidum* and *Bf. dentium* were rarely isolated (Fig. 2).

Although the aim of the study was not an in-depth characterization of the LAB, bifidobacteria and *Bacteroides* bacterial communities isolated from healthy human faeces, the microorganisms isolated were typical of this environment. Of the LAB isolated in this study, the majority of lactobacilli identified (*L. sakei, L. rhamnosus, L. acidophilus, L. curvatus*) may be considered to be of food origin and associated with westernized populations, as previously described [23]. For bifidobacteria, the most frequently isolated species were *Bf. longum, Bf. adolescentis* and *Bf. animalis*. This is in agreement with the literature, which shows that *Bf. adolescentis* and *Bf. longum* are predominant bifidobacteria in adults [24]. The *Bacteroides* spp. identified have also been described to be part of the human faecal community [25].

The non-*Bacteroides* bacteria isolated from BBE agar were *Parabacteroides distasonis, Phocaeicola vulgatus, Phocaeicola dorei* and *Coprococcus eutactus*. The non-lactobacilli isolated from MRS agar included *Alteribacillus bidgolensis, Dialister succinatiphilus, Streptococcus anginosus, Streptococcus mutans* and *Weissella viridescens*. The non-bifididobacteria isolated from LP-MRS included *Blautia obeum, Nitrosomonas oligotropha, Pantoea agglomerans* and *Ruminococcus faecis* (basonym of *Mediterraneibacter faecis*). As expected, our results show that the selective media used for the isolation of lactobacilli, bifidobacteria and *Bacteroides* are not exclusively selective and supported the growth of other faecal bacteria such as *Dialister, Alteribacillus, Blautia, Nitrosomonas, Pantoea, Coprococcus, Phocaeicola* and *Parabacteroides* spp.

**Fig. 3:**
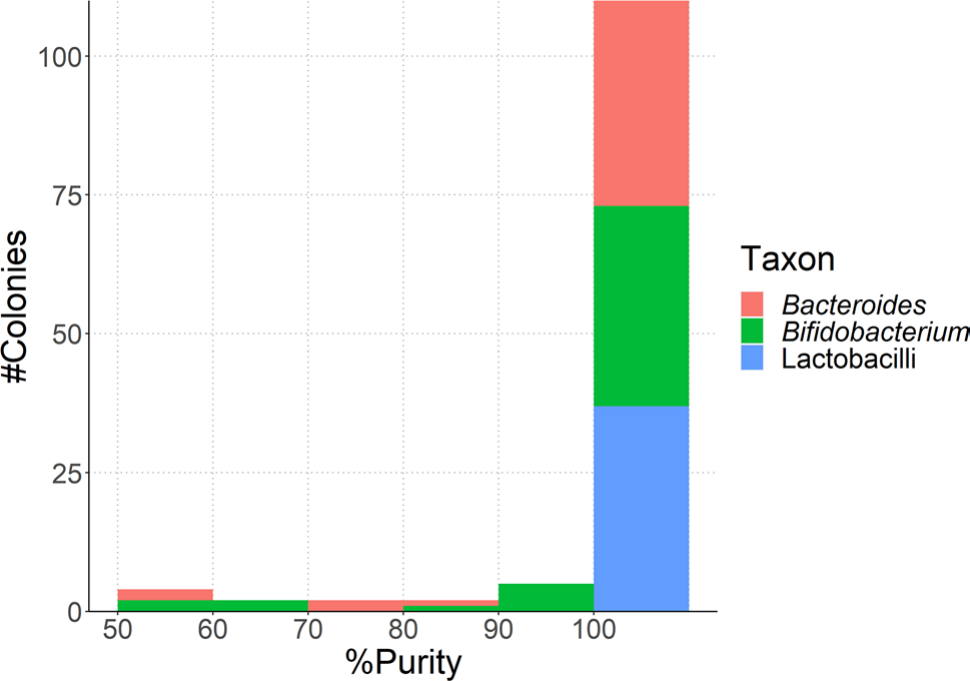
Purity of the 125 colonies that were identified as lactobacilli, bifidobacteria and *Bacteroides*. Red colour corresponds to colonies identified as *Bacteroides,* green to bifidobacteria and blue to lactobacilli (Color figure online)

**Fig. 4:**
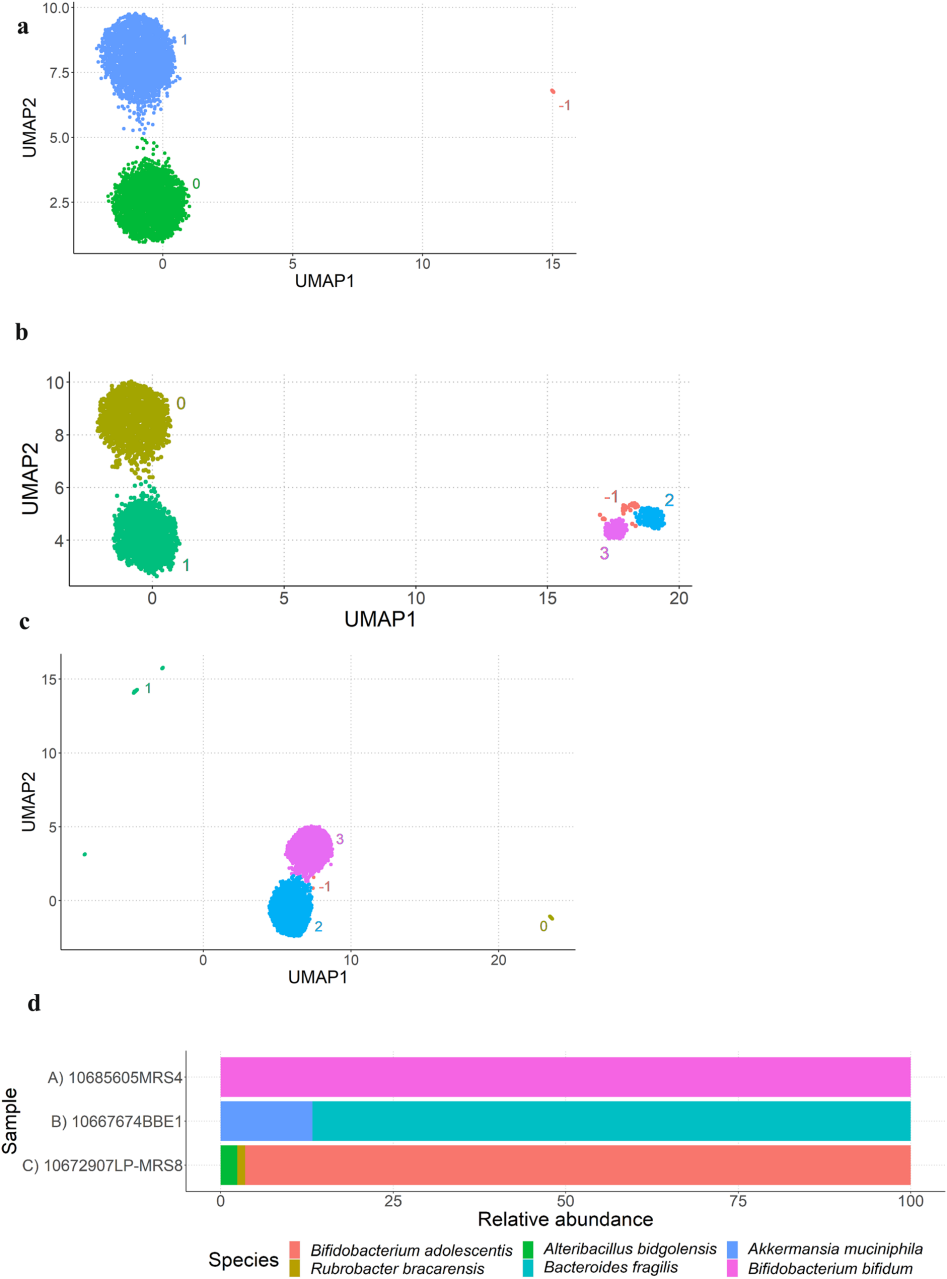
NanoCLUST plots obtained after sequencing of selected colonies showing clustering of sequence reads and read percentage. **A** Isolate 10685605MRS4, identified as *Bf. bifidum* with 100% purity, for which clusters 0 (green) and 1 (blue) were identified as *Bf. bifidum*. **B** Sample 10667674BBE1, identified as *Ba. fragilis* with 86.69% purity, for which clusters 0 (golden) and 1 (green) were identified as *Ba. fragilis* and clusters 2 (blue) and 3 (pink) as *Akkermansia muciniphila*. **C** Isolate 10672907LP-MRS8, identified as *Bf. adolescentis* with 96.48% purity, for which cluster 0 (golden) was identified as *Rubrobacter bracarensis*, cluster 1 (green) as *Alteribacillus bidgolensis* and clusters 2 (blue) and 3 (pink) as *Bf. adolescentis*. **D** Percentage of reads attributed to each species present in each isolate. Clusters named “− 1” correspond to unclassified reads that are filtered by NanoCLUST at later stages of the analysis [12] (Color figure online)

The purity of the colonies was determined by ONT MinION sequencing and defined as the percentage of reads attributed to the most frequently identified species in the colony. A colony with 100% of reads allocated to a specific species was considered as pure. This purity level was considered sufficient for deciding whether the colony should be further examined for WGS in future investigations. Of the 180 colonies investigated, 125 were identified as lactobacilli, bifidobacteria or *Bacteroides*. Out of these, 110 showed 100% purity (88%; Fig. 3, Supplementary Table 1), while 15 of the 125 sequenced colonies were < 100% pure, and for these usually a second or, in one case, a third species could be identified (Suppl. Table 1). Out of these 15 mixed colonies, four showed purity between 50 and 60%, two colonies each showed purity between 60 and 70%, 70–80% and 80–90%, and five colonies presented purity between 90 and 100% (Fig. 3). The NanoCLUST plots illustrate one isolate that was 100% pure, indicating a single species, and two isolates with less than 100% purity, in which two or even three species could be detected (Fig. 4). As examples, it is shown that sequencing reads from colony 10685605MRS4 with 100% purity could be assigned to *Bf. bifidum* only (Fig. 4A), while colony 10667674BBE1 could be identified as *Ba. fragilis* (86.69% purity) and *Akkermansia municiphila* (Fig. 4B) and colony 10672907LP-MRS8 was determined to be a mix of *Bf. adolescentis* (96.48% purity), *Rubrobacter bracarensis* and *Alteribacillus bidgolensis* (Fig. 4C). Figure 4D shows the proportions of the identified bacteria in these three examples in further detail. It should be noted that often two clusters were obtained for a single species, which correspond to the forward and reverse sequences of the almost complete 16S rRNA gene Flongle sequencing result.

In addition, we randomly selected four isolates (10670603MRS5, 10685605MRS4, 10693850MRS2 and 10693850MRS7) for precise strain identification through WGS by Illumina short-read sequencing. Strain identification was performed by calculation of the ANI and the dDDH with closely related type strains, i.e. *L. acidophilus* DSM 20079^ T^ (acc. no. CP020620), *Bf. bifidum* LMG 11041^ T^ (acc. no. JGYO01000000), *Bf. dentium* Bd1^T^ (acc. no. JDUY00000000) and *L. rhamnosus* DSM 20021^ T^ (acc. no. AZCQ01000000). When compared with the type strains, the ANI and dDDH values exceeded the cut-off values for species delineation and indicated the very same identification as NanoCLUST with ONT MinION sequencing (Supplementary Table 2 and 3, and Supplementary Fig. 1).

Our aim was to identify bacteria present in complex samples and to select specific species for which there are no exclusively selective media. Both of our goals were achieved successfully and efficiently by combining and adapting several existing technologies. First, this method requires no more than two culturing steps, namely the culturing of the samples on agar plates and the single-step culturing of the selected resulting colonies in broth medium. Second, our DNA extraction method worked for all bacterial taxa, and it took less than 25 min regardless of the number of samples (limited only by the space in the ultrasonicator) and without increasing the handling workload. This is highly advantageous compared to all other methods that we tested, especially for Gram-positive bacteria. Bead beating and heat-based techniques were unsuccessful, and kit-based extractions are more expensive and require a higher workload.

Furthermore, 16S rRNA gene sequencing has been shown to provide an accurate bacterial taxonomic classification [26]. The 16S rRNA gene is around 1550 bp, and it includes both conserved regions, which allow for the design of universal primers for different bacterial taxa, and variable regions that allow for reliable bacterial taxonomic identification [27]. Although ONT MinION sequencing is known to be error-prone, resulting in sequences of approximately 95% accuracy [28], it allows for long-read sequencing. Therefore, 16S rRNA gene sequencing using a long-read sequencing platform can provide an accurate taxonomic classification when compared to short-read platforms such as Illumina because it does not require any assembling steps. Naturally, WGS data can provide better species resolution especially between closely related species, but that would be at the expense of time, resources and costs spent on colonies that are not the focus of our future studies, such as the abundant *Enterococcus* strains that we found. Nonetheless, we performed WGS on four randomly selected isolates to check for the accuracy of the identification, and we obtained consistent results between the two techniques. Finally, NanoCLUST has outperformed state-of-the-art software both in terms of identification accuracy and abundance profile estimation at the species level when classifying mock communities [12]. This analysis pipeline is based on an unsupervised read clustering step followed by the generation of a consensus sequence, which is sufficient for accurate species identification through BLAST classification and greatly increases the speed of the pipeline compared to alternatives that perform BLAST on every read. In this way, the bacteria obtained from the colonies could be accurately identified by matching the consensus sequences to the NCBI Refseq database [12]. Additionally, NanoCLUST provides an insight into the number of reads of each cluster, which allows the user to have an overview of the purity of the isolate.

Alternative methods such as MALDI-TOF require pure colonies, costly equipment such as the MALDI-TOF mass spectrometer, and extensive databases of protein profiles, often developed in-house [6, 29]. In contrast, in this study identification could be achieved with minimal culturing, comparatively low-cost equipment and 16S rRNA gene sequencing by MinION Flongle sequencing, which is superior when compared to conventional Sanger sequencing regarding cost and time. More specifically, our method offers a cost per sample below 5€ from the beginning to the end of the pipeline, and it can take less than 2 weeks to identify 96 samples, whereas with other methods, it could take around 4 weeks due to the need for pure colonies, high-workload DNA extraction methods and slower sequencing procedures. The method described here also serves to circumvent the need for sub-culturing by allowing a rapid assessment of whether the cultures are pure for utilization in further studies. If not, it allows for decisions on whether to further purify the bacteria present in the colonies, depending on the goal of future studies, which may include taxonomical or genomic investigations, as well as investigations of functional properties. In addition, the method may be used to identify and culture specific target bacteria to be added to culture collections. The method may also present a valuable addition to future culturomics studies, possibly negating the need for MALDI-TOF MS identification techniques.

## Conclusion

The method here described allows for an accurate bacterial identification as well as an insight into the purity of the isolates. Therefore, it can be useful for having a fast, unexpensive and robust overview of the bacteria extracted from complex microbiological samples before selecting them for further studies.

## Supplementary Information

Below is the link to the electronic supplementary material.

Supplementary file1 (DOCX 70 KB)

## Data Availability

The datasets and code generated during and/or analysed during the current study are available from the corresponding author on reasonable request.
