# Supplementary material for: Fast Identification Method for Screening Bacteria from Faecal Samples Using Oxford Nanopore Technologies MinION Sequencing
Source: Curr Microbiol. 2023 Feb 9;80(3):101. doi: 10.1007/s00284-023-03201-7 (PMC9911510; doi:10.1007/s00284-023-03201-7)

**Fast Identification Method for Screening Bacteria from Faecal Samples Using Oxford Nanopore Technologies MinION Sequencing**

Ana Sofia G. Borges, Meghna Basu, Erik Brinks, Corinna Bang, Gyu-Sung Cho^*^, John F. Baines, Andre Franke and Charles M.A.P. Franz

Current Microbiology Journal

*Corresponding Author: Dr. Gyu-Sung Cho

Department of Microbiology and Biotechnology, Max Rubner-Institut, Hermann-Weigmann-Straße 1, 24103 Kiel, Germany.

Email: [Gyusung.Cho@mri.bund.de](mailto:Gyusung.Cho@mri.bund.de)

Supplementary Table 1 – Purity and contaminants of the lactobacilli, bifidobacteria and *Bacteroides* spp. cultured in this study. The “Total no. of reads” column does not include the unclassified reads.

| **Sample name** | **Most frequent species** | **No. of reads assigned to most frequent species** | **Total no. of reads** | **Purity (%)** | **Other microorganims** |
| --- | --- | --- | --- | --- | --- |
| 10685605BBE1 | *Bacteroides cellulosilyticus* | 145 | 145 | 100.00 |  |
| 10685605BBE3 | *Bacteroides cellulosilyticus* | 1132 | 1132 | 100.00 |  |
| 10667674BBE2 | *Bacteroides fragilis* | 965 | 965 | 100.00 |  |
| 10667674BBE3 | *Bacteroides fragilis* | 875 | 875 | 100.00 |  |
| 10667674BBE4 | *Bacteroides fragilis* | 928 | 928 | 100.00 |  |
| 10667674BBE5 | *Bacteroides fragilis* | 985 | 985 | 100.00 |  |
| 10667674BBE9 | *Bacteroides fragilis* | 973 | 973 | 100.00 |  |
| 10667674BBE10 | *Bacteroides fragilis* | 1047 | 1047 | 100.00 |  |
| 10685605BBE2 | *Bacteroides fragilis* | 1068 | 1068 | 100.00 |  |
| 10685605BBE5 | *Bacteroides fragilis* | 424 | 424 | 100.00 |  |
| 10685605BBE6 | *Bacteroides fragilis* | 1035 | 1035 | 100.00 |  |
| 10685605BBE7 | *Bacteroides fragilis* | 593 | 593 | 100.00 |  |
| 10670603BBE1 | *Bacteroides ovatus* | 1045 | 1045 | 100.00 |  |
| 10685605BBE4 | *Bacteroides ovatus* | 1249 | 1249 | 100.00 |  |
| 10685605BBE8 | *Bacteroides ovatus* | 949 | 949 | 100.00 |  |
| 10685605BBE9 | *Bacteroides ovatus* | 1115 | 1115 | 100.00 |  |
| 10685605BBE10 | *Bacteroides ovatus* | 1173 | 1173 | 100.00 |  |
| 10670603BBE5 | *Bacteroides thetaiotaomicron* | 895 | 895 | 100.00 |  |
| 10672907BBE1 | *Bacteroides thetaiotaomicron* | 1253 | 1253 | 100.00 |  |
| 10672907BBE2 | *Bacteroides thetaiotaomicron* | 1220 | 1220 | 100.00 |  |
| 10672907BBE8 | *Bacteroides thetaiotaomicron* | 1102 | 1102 | 100.00 |  |
| 10672907BBE10 | *Bacteroides thetaiotaomicron* | 1051 | 1051 | 100.00 |  |
| 10670603BBE4 | *Bacteroides uniformis* | 1104 | 1104 | 100.00 |  |
| 10670603BBE6 | *Bacteroides uniformis* | 1129 | 1129 | 100.00 |  |
| 10672907BBE3 | *Bacteroides uniformis* | 806 | 806 | 100.00 |  |
| 10672907BBE4 | *Bacteroides uniformis* | 949 | 949 | 100.00 |  |
| 10672907BBE6 | *Bacteroides uniformis* | 640 | 640 | 100.00 |  |
| 10692698BBE1 | *Bacteroides xylanisolvens* | 854 | 854 | 100.00 |  |
| 10692698BBE2 | *Bacteroides xylanisolvens* | 1179 | 1179 | 100.00 |  |
| 10692698BBE3 | *Bacteroides xylanisolvens* | 1007 | 1007 | 100.00 |  |
| 10692698BBE4 | *Bacteroides xylanisolvens* | 1221 | 1221 | 100.00 |  |
| 10692698BBE5 | *Bacteroides xylanisolvens* | 1104 | 1104 | 100.00 |  |
| 10692698BBE6 | *Bacteroides xylanisolvens* | 1193 | 1193 | 100.00 |  |
| 10692698BBE7 | *Bacteroides xylanisolvens* | 1349 | 1349 | 100.00 |  |
| 10692698BBE8 | *Bacteroides xylanisolvens* | 879 | 879 | 100.00 |  |
| 10692698BBE9 | *Bacteroides xylanisolvens* | 957 | 957 | 100.00 |  |
| 10692698BBE10 | *Bacteroides xylanisolvens* | 954 | 954 | 100.00 |  |
| 10667674MRS3 | *Bifidobacterium adolescentis* | 923 | 923 | 100.00 |  |
| 10672907LP-MRS10 | *Bifidobacterium adolescentis* | 1026 | 1026 | 100.00 |  |
| 10672907MRS4 | *Bifidobacterium adolescentis* | 3173 | 3173 | 100.00 |  |
| 10672907MRS5 | *Bifidobacterium adolescentis* | 444 | 444 | 100.00 |  |
| 10672907MRS8 | *Bifidobacterium adolescentis* | 3454 | 3454 | 100.00 |  |
| 10667674LP-MRS3 | *Bifidobacterium animalis* | 582 | 582 | 100.00 |  |
| 10667674LP-MRS7 | *Bifidobacterium animalis* | 765 | 765 | 100.00 |  |
| 10667674LP-MRS10 | *Bifidobacterium animalis* | 2049 | 2049 | 100.00 |  |
| 10667674MRS1 | *Bifidobacterium animalis* | 381 | 381 | 100.00 |  |
| 10667674MRS5 | *Bifidobacterium animalis* | 2347 | 2347 | 100.00 |  |
| 10667674MRS8 | *Bifidobacterium animalis* | 1029 | 1029 | 100.00 |  |
| 10667674LP-MRS4 | *Bifidobacterium bifidum* | 6907 | 6907 | 100.00 |  |
| 10667674MRS7 | *Bifidobacterium bifidum* | 1252 | 1252 | 100.00 |  |
| 10685605MRS4 | *Bifidobacterium bifidum* | 5147 | 5147 | 100.00 |  |
| 10693850MRS2 | *Bifidobacterium dentium* | 4890 | 4890 | 100.00 |  |
| 10693850MRS6 | *Bifidobacterium dentium* | 4981 | 4981 | 100.00 |  |
| 10672907LP-MRS4 | *Bifidobacterium longum* | 1084 | 1084 | 100.00 |  |
| 10672907LP-MRS9 | *Bifidobacterium longum* | 1170 | 1170 | 100.00 |  |
| 10672907MRS6 | *Bifidobacterium longum* | 5237 | 5237 | 100.00 |  |
| 10672907MRS9 | *Bifidobacterium longum* | 2087 | 2087 | 100.00 |  |
| 10685605LP-MRS1 | *Bifidobacterium longum* | 934 | 934 | 100.00 |  |
| 10685605LP-MRS2 | *Bifidobacterium longum* | 1383 | 1383 | 100.00 |  |
| 10685605LP-MRS3 | *Bifidobacterium longum* | 1475 | 1475 | 100.00 |  |
| 10685605LP-MRS4 | *Bifidobacterium longum* | 1672 | 1672 | 100.00 |  |
| 10685605LP-MRS5 | *Bifidobacterium longum* | 1122 | 1122 | 100.00 |  |
| 10685605LP-MRS6 | *Bifidobacterium longum* | 434 | 434 | 100.00 |  |
| 10685605LP-MRS8 | *Bifidobacterium longum* | 1516 | 1516 | 100.00 |  |
| 10685605LP-MRS9 | *Bifidobacterium longum* | 952 | 952 | 100.00 |  |
| 10685605MRS1 | *Bifidobacterium longum* | 3924 | 3924 | 100.00 |  |
| 10685605MRS3 | *Bifidobacterium longum* | 1603 | 1603 | 100.00 |  |
| 10685605MRS5 | *Bifidobacterium longum* | 3344 | 3344 | 100.00 |  |
| 10685605MRS7 | *Bifidobacterium longum* | 2528 | 2528 | 100.00 |  |
| 10685605MRS9 | *Bifidobacterium longum* | 7291 | 7291 | 100.00 |  |
| 10685605MRS10 | *Bifidobacterium longum* | 1833 | 1833 | 100.00 |  |
| 10693850LP-MRS2 | *Bifidobacterium longum* | 1362 | 1362 | 100.00 |  |
| 10693850LP-MRS9 | *Bifidobacterium longum* | 521 | 521 | 100.00 |  |
| 10693850LP-MRS7 | *Lacticaseibacillus rhamnosus* | 1689 | 1689 | 100.00 |  |
| 10693850MRS7 | *Lacticaseibacillus rhamnosus* | 5781 | 5781 | 100.00 |  |
| 10670603MRS1 | *Lactobacillus acidophilus* | 3997 | 3997 | 100.00 |  |
| 10670603MRS2 | *Lactobacillus acidophilus* | 5691 | 5691 | 100.00 |  |
| 10670603MRS3 | *Lactobacillus acidophilus* | 3735 | 3735 | 100.00 |  |
| 10670603MRS5 | *Lactobacillus acidophilus* | 6034 | 6034 | 100.00 |  |
| 10670603MRS6 | *Lactobacillus acidophilus* | 4900 | 4900 | 100.00 |  |
| 10670603MRS9 | *Lactobacillus acidophilus* | 5484 | 5484 | 100.00 |  |
| 10670603LP-MRS1 | *Latilactobacillus curvatus* | 4785 | 4785 | 100.00 |  |
| 10670603LP-MRS3 | *Latilactobacillus curvatus* | 5461 | 5461 | 100.00 |  |
| 10670603LP-MRS5 | *Latilactobacillus curvatus* | 4511 | 4511 | 100.00 |  |
| 10692698MRS4 | *Latilactobacillus curvatus* | 5050 | 5050 | 100.00 |  |
| 10670603LP-MRS2 | *Latilactobacillus sakei* | 1651 | 1651 | 100.00 |  |
| 10670603LP-MRS4 | *Latilactobacillus sakei* | 4981 | 4981 | 100.00 |  |
| 10670603LP-MRS7 | *Latilactobacillus sakei* | 5577 | 5577 | 100.00 |  |
| 10670603LP-MRS8 | *Latilactobacillus sakei* | 5178 | 5178 | 100.00 |  |
| 10670603LP-MRS9 | *Latilactobacillus sakei* | 5239 | 5239 | 100.00 |  |
| 10670603LP-MRS10 | *Latilactobacillus sakei* | 4854 | 4854 | 100.00 |  |
| 10670603MRS4 | *Latilactobacillus sakei* | 4124 | 4124 | 100.00 |  |
| 10670603MRS7 | *Latilactobacillus sakei* | 6276 | 6276 | 100.00 |  |
| 10692698LP-MRS1 | *Latilactobacillus sakei* | 5554 | 5554 | 100.00 |  |
| 10692698LP-MRS2 | *Latilactobacillus sakei* | 4703 | 4703 | 100.00 |  |
| 10692698LP-MRS3 | *Latilactobacillus sakei* | 5498 | 5498 | 100.00 |  |
| 10692698LP-MRS4 | *Latilactobacillus sakei* | 5175 | 5175 | 100.00 |  |
| 10692698LP-MRS5 | *Latilactobacillus sakei* | 5147 | 5147 | 100.00 |  |
| 10692698LP-MRS6 | *Latilactobacillus sakei* | 5265 | 5265 | 100.00 |  |
| 10692698LP-MRS7 | *Latilactobacillus sakei* | 4826 | 4826 | 100.00 |  |
| 10692698MRS1 | *Latilactobacillus sakei* | 4400 | 4400 | 100.00 |  |
| 10692698MRS2 | *Latilactobacillus sakei* | 5100 | 5100 | 100.00 |  |
| 10692698MRS3 | *Latilactobacillus sakei* | 4394 | 4394 | 100.00 |  |
| 10692698MRS5 | *Latilactobacillus sakei* | 4621 | 4621 | 100.00 |  |
| 10692698MRS6 | *Latilactobacillus sakei* | 4725 | 4725 | 100.00 |  |
| 10692698MRS7 | *Latilactobacillus sakei* | 4164 | 4164 | 100.00 |  |
| 10692698MRS8 | *Latilactobacillus sakei* | 4264 | 4264 | 100.00 |  |
| 10692698MRS9 | *Latilactobacillus sakei* | 4324 | 4324 | 100.00 |  |
| 10685605LP-MRS7 | *Ligilactobacillus ruminis* | 1882 | 1882 | 100.00 |  |
| 10685605MRS8 | *Ligilactobacillus ruminis* | 5183 | 5183 | 100.00 |  |
| 10672907MRS2 | *Bifidobacterium adolescentis* | 4972 | 5024 | 98.96 | *Ruminococcus faecis* |
| 10685605MRS2 | *Bifidobacterium longum* | 5179 | 5250 | 98.65 | *Abiotrophia defectiva* |
| 10667674MRS10 | *Bifidobacterium animalis* | 5211 | 5349 | 97.42 | *Alteribacillus alkaliphilus* |
| 10672907MRS7 | *Bifidobacterium adolescentis* | 4839 | 4982 | 97.13 | *Ruminococcus faecis* |
| 10672907LP-MRS8 | *Bifidobacterium adolescentis* | 6488 | 6725 | 96.48 | *Alteribacillus bidgolensis; Rubrobacter bracarensis* |
| 10672907LP-MRS2 | *Bifidobacterium longum* | 1223 | 1383 | 88.43 | *Bifidobacterium adolescentis* |
| 10667674BBE1 | *Bacteroides fragilis* | 2710 | 3126 | 86.69 | *Akkermansia muciniphila* |
| 10667674BBE6 | *Bacteroides caccae* | 626 | 810 | 77.28 | *Bacteroides fragilis* |
| 10667674BBE7 | *Bacteroides fragilis* | 688 | 967 | 71.15 | *Bacteroides caccae* |
| 10672907MRS10 | *Bifidobacterium adolescentis* | 3049 | 4406 | 69.20 | *Pantoea allii* |
| 10685605LP-MRS10 | *Bifidobacterium adolescentis* | 760 | 1249 | 60.85 | *Enterococcus faecium* |
| 10667674BBE8 | *Bacteroides caccae* | 552 | 927 | 59.55 | *Ruminococcus torques* |
| 10670603BBE9 | *Bacteroides uniformis* | 655 | 1140 | 57.46 | *Lawsonibacter asaccharolyticus* |
| 10667674LP-MRS8 | *Bifidobacterium adolescentis* | 3897 | 6835 | 57.02 | *Bifidobacterium bifidum* |
| 10672907LP-MRS7 | *Bifidobacterium adolescentis* | 458 | 905 | 50.61 | *Bifidobacterium longum* |

Supplementary Table 2 – dDDH analysis.

|  |  | **Formula 2** |  |  |  | **G+C difference** |
| --- | --- | --- | --- | --- | --- | --- |
| **Query genome** | **Reference genome** | **DDH** | **Model C.I.** | **Distance** | **Prob. DDH >= 70%** |  |
| *Lacticaseibacillus* 10693850MRS7 | *Lacticaseibacillus rhamnosus* DSM 20021^T^ | 96.1 | [94.8 - 97.2%] | 0.0053 | 97.46 | 0.1 |
|  |  |  |  |  |  |  |
|  |  | **Formula 2** |  |  |  | **G+C difference** |
| **Query genome** | **Reference genome** | **DDH** | **Model C.I.** | **Distance** | **Prob. DDH >= 70%** |  |
| *Lactobacillus* 10670603MRS5 | *Lactobacillus acidophilus* DSM20079^T^ | 99.60 | [99.3 - 99.7%] | 0.0009 | 98.19 | 0.14 |
|  |  |  |  |  |  |  |
|  |  | **Formula 2** |  |  |  | **G+C difference** |
| **Query genome** | **Reference genome** | DDH | **Model C.I.** | **Distance** | **Prob. DDH >= 70%** |  |
| *Bifidobacterium* 10693850MRS2 | *Bif. dentium* Bd1^T^ | 90.80 | [88.6 - 92.6%] | 0.0112 | 96.06 | 0.06 |
|  |  |  |  |  |  |  |
|  |  | **Formula 2** |  |  |  | **G+C difference** |
| **Query genome** | **Reference genome** | DDH | **Model C.I.** | **Distance** | **Prob. DDH >= 70%** |  |
| *Bifidobacterium* 10685605MRS4 | *Bif. bifidum* LMG 11041^T^ | 93.30 | [91.4 - 94.8%] | 0.0086 | 96.77 | 0.06 |

Supplementary Table 3 – Precise strain identification with ANI and dDDH.

| **Isolate** | **Type strain** | **ANI (%)** | **dDDH formula 2 (%)** |
| --- | --- | --- | --- |
| 10685605MRS4 | *Bif. bifidum* LMG 11041^T^ | 99.21 | 93.3 |
| 10693850MRS2 | *Bif. dentium* Bd1^T^ | 98.8 | 90.8 |
| 10693850MRS7 | *L. rhamnosus* DSM 20021^T^ | 99.49 | 96.1 |
| 10670603MRS5 | *L. acidophilus* DSM 20079^T^ | 99.98 | 99.6 |

Supplementary Figure 1 – ANI heatmap.


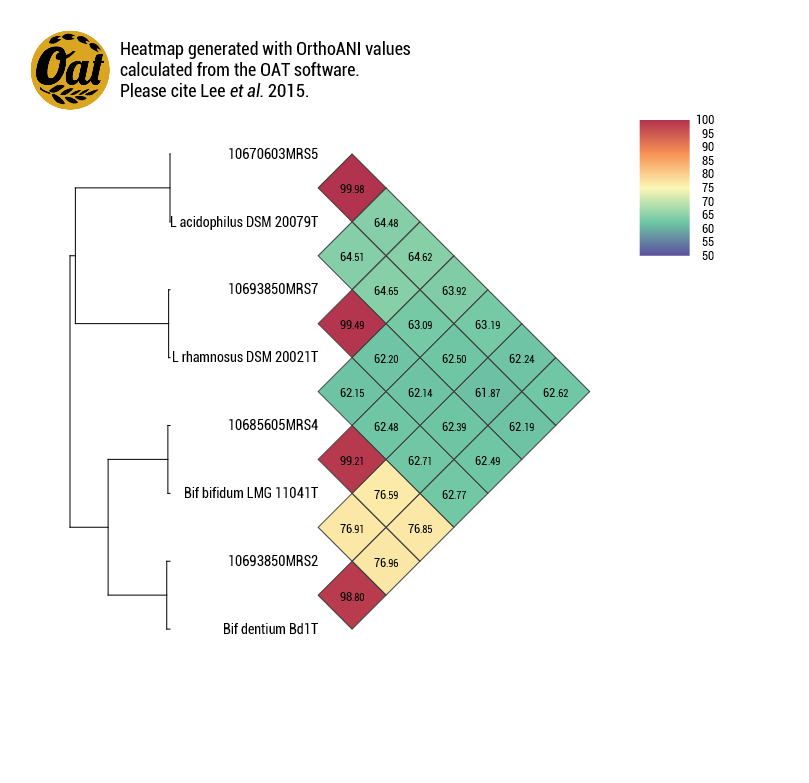

Supplement: Supplementary file 1 — Supplementary file1 (DOCX 70 KB) [file 284_2023_3201_MOESM1_ESM.docx]
